# A Nurse-Led Intervention in General Practice to Manage People with Chronic Conditions: A Protocol for a Quasi-Experimental Study

**DOI:** 10.3390/healthcare14131830

**Published:** 2026-06-24

**Authors:** Federica Canzan, Jessica Longhini, Michela Filippi, Giulia Marini, Chiara Leardini, Achille Di Falco, Elisa Ambrosi

**Affiliations:** 1 Department of Diagnostic and Public Health, University of Verona, 37134 Verona, Italy; michela.filippi_02@univr.it (M.F.); elisa.ambrosi_01@univr.it (E.A.); 2 Department of Cardio-Thoracic-Vascular Sciences and Public Health, University of Padua, 35131 Padova, Italy; jessica.longhini@unipd.it; 3 Department of Human Science, University of Verona, 37134 Verona, Italy; giulia.marini_02@univr.it; 4 Department of Management, University of Verona, 37134 Verona, Italy; chiara.leardini@univr.it; 5 Social Health Services, ULSS 8 Berica, 36100 Vicenza, Italy; achille.difalco@aulss8.veneto.it

**Keywords:** general practice, primary care, nurses, chronic disease

## Abstract

**Background/Objectives**: Chronic diseases account for 74% of global deaths, with multimorbidity (existence of more than one chronic condition) increasing disability risk and treatment burden, leading to poor adherence, disease progression, and reduced quality of life. Nursing-led proactive care models that focus on patient engagement, education, and self-care can help mitigate these challenges. The study aims to evaluate the effectiveness of a nurse-led proactive health intervention in improving care for individuals with chronic diseases in general practice. **Methods**: A quasi-experimental pre–post study will be conducted in a Community Health Home in Northern Italy. Family and community nurses will deliver the intervention, which includes assessments, educational sessions, and follow-ups for patients aged 65+ with at least one chronic condition. Recruitment will occur over three months. **Results**: Primary outcomes include emergency department visits and hospitalizations, while secondary outcomes focus on medication adherence, self-care, and service utilization. Data will be collected at 6 and 12 months, and statistical analysis will use descriptive methods and generalized estimating equations (GEEs). **Conclusions**: This study will improve the understanding of the value of nurse-led proactive intervention, filling the gap in the literature by testing evidence-based approaches on a realistic frail population. Moreover, delivering a complex but structured intervention will provide evidence for future interventions to reduce treatment burden and improve health outcomes.

## 1. Introduction

Population aging and socio-environmental changes have globally led to an increase in conditions like hypertension, overweight, obesity, and physical inactivity, identified as significant risk factors for the development of long-term diseases and potentially fatal acute events [1]. Chronic diseases are responsible for nearly 74% of global deaths [1]. In addition, multimorbidity significantly increases the risk of disability [2]. Nonetheless, multimorbidity can lead to a treatment burden, which, in turn, can result in non-adherence to treatment, disease progression, poor health status and quality of life, and caregiver burden [3]. These consequences of chronic diseases call for interventions to prevent and reduce multimorbidity and the related treatment burden by promoting proactive care for community-dwelling people [3]. These proactive health interventions involve engaging with populations, communities, families, and individuals to foster empowerment and self-care skills, defined as the ability of individuals, families, and communities to promote health, prevent disease, maintain health, and cope with illness and disability. Proactive healthcare has been widely promoted and implemented internationally, primarily through the development and adoption of innovative community and primary care models, such as the Chronic Care Model [4]. This model comprises six components: a proactive care approach that promotes patient engagement and the development of empowerment skills; the involvement of community resources; structural changes within healthcare organizations; the implementation of multidisciplinary teams; and the use of digital systems to support patient care [5]. This is supported by evidence-based decision-making and a patient-centered care model [4]. The model has been widely applied in primary care and is considered the first element of a continuous care process and the first point of contact for individuals, families, and communities within Campo’s healthcare system [6]. The World Health Organization supports the development of these types of models and their global spread. Indeed, the Alma-Ata Declaration in 1978 and the renewed Astana Declaration in 2018 [7] emphasize the importance of primary care to promote sustainable prevention and proactive health interventions, thereby reducing the occurrence of multimorbidity and disability. In this context, the nursing role is crucial for proactively and effectively responding to the needs of individuals, families, and communities through interventions focused on prevention, health promotion, and education [8,9]. Various systematic reviews highlight the effectiveness of nurse-led care models in primary care, reporting significant improvements in treatment adherence, self-care, self-efficacy, quality of life, and reduced incidence of complications [10,11,12]. These models have primarily been studied in specific population groups, involving frail individuals [13] with chronic conditions such as COPD [14], hypertension, diabetes, and cardiovascular diseases [15,16,17], as well as those exposed to risk factors like smoking habits [18]. Therefore, although a substantial body of literature demonstrates the effectiveness of nurse-led care models in primary care settings, there is no evidence on the impact of proactive nursing care on specific conditions in primary care. This could provide evidence of a nurse-led intervention that is adequate across various chronic conditions, improving outcomes for individuals with multiple chronic conditions while assessing its effect on particular multimorbidity patterns [3].

Therefore, this study aims to evaluate the effectiveness of a nurse-led, proactive health intervention focused on educational care for individuals with chronic diseases in primary care.

## 2. Methods

### 2.1. Study Design and Setting

A quasi-experimental pre–post study will be conducted in a Community Health Home in a rural district in the North of Italy with a population of 15,917 citizens, served by one local hospital. The Community Health Home is a recently introduced service in Italy, mandated by the National Law, to improve the quality of primary care. It includes different professionals within the same community structure, such as general practitioners, specialist physicians, social workers, and other allied health professionals, as well as home care nursing services and family and community nursing services. Specifically, this study directly involves this last group of professionals. Family and community nurses will deliver a structured educational intervention to outpatients with chronic diseases, aiming to reduce unhealthy lifestyles and harmful health behaviors and to promote health in the community. In the Community Health Home setting of the study, two family and community nurses with a Master of Science in primary care work from 7.00 a.m. to 3.00 p.m.

The study has been registered with the number NCT07479810 on https://clinicaltrials.gov/ (accessed on 18 March 2026).

### 2.2. Population

We will include all patients over 65 years old, assigned to general practitioners in the district of reference of the Community Health Home, diagnosed with at least one non-oncological chronic condition. Exclusion criteria are a life expectancy of less than 12 months, as assessed by the general practitioner, and an active plan for intensive home care (more than one home access/week). Potential participants will be referred by general practitioners, social workers, or other professionals from home care, community, and transitional care services to family and community nurses (hereafter, nurses). They will ascertain the inclusion criteria and collect informed written consent to participate in the study. Patients assigned to nurses will be recruited through consecutive sampling. It is not possible to perform an a priori power analysis because no data are available on the composite primary endpoint. However, using empirical data collected in the same setting and by estimating a difference between pre and post evaluations consistent with that observed in other similar studies [19,20], we aim to enroll 100–120 patients. Considering this information and the usual caseload of eligible users, the researchers have set a 3-month enrollment period.

### 2.3. Procedure

**Figure 1:**
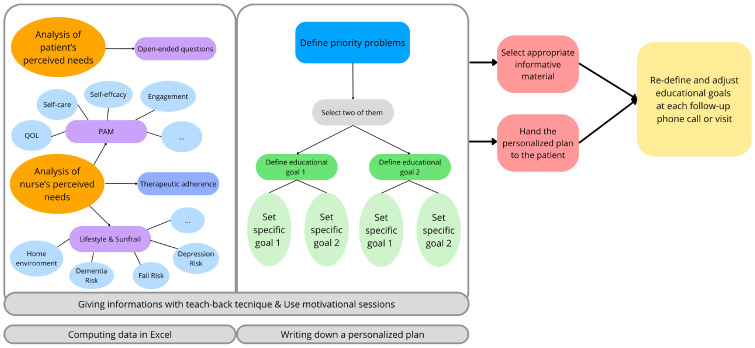
The figure illustrates the educational pathway of the intervention, detailed below. The first box represents the initial assessment, followed by the definition of problems and goal setting. Through the process, nurses will collect data and also carry on the educational intervention with multiple techniques. The resulting tailored plan will be monitored and improved through follow-up sessions.

A logic model [21,22] has been developed to organize the intervention’s components and guide the development of the protocol (Figure 1). The model was developed following a systematic review of effective nurse-led interventions to manage chronic conditions in primary care [23] and a local qualitative study of patients with multimorbidity and their informal caregivers regarding their preferences and needs for current primary care nursing services. After a six-hour training program conducted by researchers across three sessions, nurses will deliver the experimental care to patients with chronic conditions. The intervention is rooted in the Chronic Care Model [4,24] and the Middle Range Theory of self-care in chronic illnesses [25,26]. This proactive approach will be proposed and explained to patients through an initial phone call. On this occasion, users who agree to participate will be scheduled for an appointment or a home visit, depending on their ability to reach the Community Health Home. Then, nurses will conduct an initial structured assessment with interviews and tools, and will set follow-ups to monitor self-care development and clinical conditions. Patients will receive at least three contacts over six months, alternating between in-person visits and phone calls. However, nurses can increase contact frequency when patients present with multiple educational needs or poor self-care skills.

### 2.4. Intervention

The intervention will be the only manipulated variable, as the nurses involved are the same that would have been leading care for patients and their informal caregivers within the Community Home. Nurses will conduct a therapeutic education program following standard components developed based on multiple frameworks, theories, and evidence, focusing on behavioral changes [27], enhancing engagement and empowerment [28,29], self-care and symptom management [25,26,30], and planning health promotion strategies [31].

Specifically, during the first interview, the nurse will collect the following data.

(a) Socio-demographic status and family network: age, sex, cohabitation status and designated caregiver(s).
(b) Patient and caregiver’s priority needs, capturing concerns, difficulties, and expectations with open-ended questions.
(c) Clinical condition: vital signs, BMI, diseases and related medications, civil disability status, medical exams, and blood tests.
(d) Socio-educational needs, meaning the following:
  - Lifestyles, combining physical activity, sleeping quality, alcohol consumption, and smoking habits;
  - Self-care skills, using the Patient Activation Measure (PAM) questionnaire [32];
  - Clinical–social frailty, assessing the risk with the Sunfrail questionnaire [33].

Other specific questionnaires will be used whenever concerns or issues are faced. All questionnaires that will be administered are validated in Italian; for each patient, scores will be collected in a specifically designed Excel file. This spreadsheet will serve as electronic documentation, including questionnaires, automated scoring, a summary of active clinical and educational issues, a medication plan, and goals shared with the patient. These elements will be prefilled based on nurses’ data input and finalized manually. The tool will serve as a guide for nurses to deliver the intervention and will become part of the healthcare electronic system. All information will be incorporated into an educational plan, which will be provided to the patient in written form.

Throughout all interviews, four communication approaches will be implemented:

(a) Teach-back [34,35]: This is a communication strategy designed to assess an individual’s understanding of newly acquired information. After presenting the information incrementally, the individual is asked to summarize what has been understood using their own words, thereby identifying any knowledge gaps. This approach actively engages the participant and holds the professional accountable for effective communication, ensuring that information is conveyed in small, manageable portions and comprehension is verified at each step.
(b) Motivational Interviewing [36,37]: This is an evidence-based counseling style that facilitates a collaborative dialogue within a supportive and empathetic environment. This approach aims to elicit the individual’s intrinsic reasons for change and strengthen their commitment and motivation to achieve specific goals. The primary components include reflective listening, open-ended questions to elicit change-oriented statements, synthesis and support affirmations, and targeted information.
(c) Sharing Evidence Routine for a Person-centered Plan for Action (SHERPA model) [38,39]: This is a structured three-phase shared planning approach. In Phase 1 (Share), the individual’s problems are identified collaboratively, including illnesses related to physical and emotional elements (diagnosis, behaviors, symptoms, concerns, emotions), social issues such as isolation or poverty, and challenges in disease management, aiming to create a person-centered problem framework rather than a diagnosis-focused one. In Phase 2 (Link), the professional and the patient discuss how to connect these issues, mapping them visually in circles of varying sizes proportional to their significance. Pathophysiological connections help the individual understand their condition, while the patient’s connections provide insights into personal values, beliefs, and non-clinical expectations. In Phase 3 (Plan), the professional and the individual prioritize these issues and set specific goals, such as reducing medication, managing symptoms, modifying unhealthy behaviors, or achieving social objectives.
(d) Interviews with family units, inspired by the Calgary Family Assessment and Intervention Models [40]: These shift the focus away from the individual patient, are designed to support families as a unit, and are particularly effective when families experience physical and emotional distress and face challenges in response to stressful events such as death, end-of-life care, or acute and chronic illnesses. By employing a systematic approach to family assessment—investigating structural, developmental, and functional categories, along with genograms and ecomaps—the nurse can identify family strengths and resources within the caregiver network and formulate appropriate interventions. Such interventions may include providing information and perspectives, using questions to promote change in the affective, behavioral, and cognitive domains of family functioning, encouraging family members to assume or relinquish caregiving roles, and activating community and social resources to support the family.

### 2.5. Outcomes

The primary clinical outcome will be a composite of at least one event, including emergency department visits or hospitalization for related pathological causes. This outcome will be evaluated pre- and post-intervention to assess the effectiveness of the proactive care on hard endpoints. On the other hand, secondary outcomes will be assessed after the intervention in terms of the following:

- Medication adherence through the calculation of the periods elapsed between prescriptions;
- Self-care ability assessed using the Patient Activation Measure (PAM) [41];
- Quality of received care by Patient-Reported Experience Measurement (PREMsT questionnaire).

The following process evaluations will also be collected during the reference period:

- Number of patients with periodic use of the nursing service (at least three visits);
- Number of nursing visits for each user;
- Number of nurse visits at home;
- Number of users that received structured family assessment interviews;
- Number of medical visits by the general practitioner for each user;
- Rate of users with new prescriptions of aids/devices;
- Number of services activated per person: physiotherapist, palliative care, social worker, etc.;
- Rate of users reported to the general practitioner for suspected exacerbation of chronic pathology.

### 2.6. Data Collection

All data needed for the analysis, meaning outcome measures, socio-demographic data, and assessment evaluations, will be computed pseudonymized in a different Excel file, secured by the research network. Information will be collected from nurses’ Excel charts and databases already in use by the pharmacy service and hospital facilities. The region shares an integrated electronic database that provides access to data from all surrounding facilities. Data collection will be carried out for the primary outcome over 12 months before and over 12 months after enrollment, and for secondary outcomes through 6 and 12 months following enrollment (Table 1). Specifically, questionnaires on self-care and the quality of received care will be administered at three time points: enrolment, 6 months, and 12 months. To establish the correlation between hospitalization and patients’ underlying chronic diseases, hospital discharge records, IDC codes, and discharge summaries will be assessed. When ICD codes do not specifically refer to “exacerbation” or “acute on chronic” or “decompensated” condition, the discharge letters will be analyzed, searching for the same information. When these criteria are not met, hospitalization will be considered generic, and two GPs will be involved to assess the correlation; any disagreement will be resolved through discussion.

### 2.7. Intervention Fidelity and Contamination

The intervention plans to provide patients with an additional service to the usual care delivered by their primary family and community nurses. For this reason, patients’ study dropout is expected to be low. Instead, the fidelity of the intervention needs to be monitored. A researcher will be responsible for overseeing the monitoring of the entire study process. Participating nurses will record the number, duration, and dates of face-to-face sessions and follow-up phone calls. To ensure adherence to the structured assessment and communication techniques, 15% of sessions will be audiotaped, contingent on participant consent. These will be reviewed using a checklist developed by the research team. The principal investigator will conduct weekly meetings with the intervention nurses to discuss their experiences, address challenges encountered during the delivery session, and provide feedback. In addition, the researcher will review the patients’ Excel files weekly. If deviations from the study protocol are identified, the nurses may be provided with further training.

### 2.8. Statistical Analysis

Sample characteristics will be synthesized after performing descriptive analysis and normality tests. Frequencies and percentages will be used for categorical variables, and means and standard deviations will be used for continuous normally distributed variables. For continuous data exhibiting skewed distributions, medians and interquartile ranges will be computed. The distributions of outcome variables will be assessed, and normalization transformations will be applied where necessary. To compare pre- and post-intervention outcomes, the Wilcoxon signed-rank test and McNemar’s test will be used. Even if the intervention is non-harmful and expectations are for a low-loss follow-up, to assess primary and secondary outcomes at the individual level, researchers will use an intention-to-treat approach. Changes in measures assessed over time will be evaluated using generalized estimating equation (GEE) models, with adjustments for baseline confounders. Statistical significance will be set at *p* < 0.05. Researchers will consider performing sensitivity analysis to enhance the transparency of the results [37]. If an external shock occurs, post hoc sensitivity analysis will be used. All analyses will be performed by the authors using R-4.5.3. and STATA 18 software.

## 3. Limitations and Transparency

This study, like many other pre–post studies, lacks a counterfactual comparison group [42,43], leaving the outcomes exposed to uncontrolled confounders and secular trends. The chosen design aims to assess the pragmatic effectiveness of a nurse-led proactive health intervention in a real-world setting. Health Community Homes are a newly established reality in Italy, designed appropriately to provide the best care for fragile patients. The eligible population and the number of users employed in this setting are nowadays specifically tailored for intercepting and designing new models to care for the elderly with almost one chronic pathology. For these reasons, using a different care facility or primary healthcare professional to provide a comparison is unfeasible. Even if the study employs a double follow-up period and logistic regression to assess confounders, this remains a major limitation for making inferential conclusions. Moreover, the study lacks a justification for the sample size. The selection of this composite endpoint is meaningful in practice, but it does not improve statistical power evaluations. The lack of formally available data makes it impossible to compute a formal sample size, even for the most frequent outcome. Although some evaluations on the cusp of statistical acceptability have been conducted, the researchers prefer not to define this as an a priori power analysis due to its poor reliability. Thereby, they chose to statistically underestimate future results to preserve the study’s informational value [44,45]. Nevertheless, post hoc power analysis would have been subject to excessive variability [46] and would not be helpful overall. Given the risk of type II error, there is no ambition to make inferential conclusions. The focus of this project is on the true value of nursing interventions, so *p* values can be considered less meaningful than other measurements [47]. Taking this into account, the results will cautiously discuss the effect size observed alongside the process evaluations of the intervention, aiming to provide policymakers with useful information on the early introduction of Community Health Homes and Family Nursing.

## Figures and Tables

**Table 1:** Outcomes and data collection points.

	T-1 (12 Months Before Enrolment)	T0 (Enrolment)	T1 (6 Months)	T2 (12 Months)
Composite outcome (access ED or hospitalization)	PRE 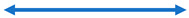	POST 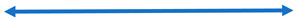		
Medication adherence (Periods elapsed between prescriptions)		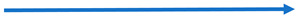		
Self-care ability assessed (PAM questionnaire)		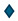	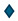	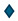
Quality of received care (PREMsT questionnaire)			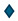	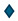
N. patients with a periodic use of nursing service (≥3 visits)		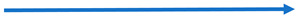		
N. of nursing visits for each user		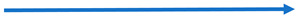		
N. of medical visits by the general practitioner for each user		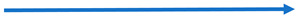		
N. of nurse visits at home		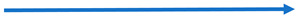		
N. of users with family assessment interviews		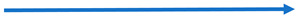		
Rate of users with new prescriptions of aids/devices		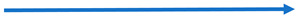		
N. of services per person (physiotherapist, social worker, etc.)		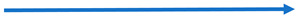		
N. of users reported to the general practitioner for suspected exacerbation		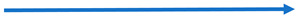		

## Data Availability

The original contributions presented in this study are included in the article. Further inquiries can be directed to the corresponding author.

## Abbreviations

The following abbreviations are used in this manuscript:

GEE	Generalized Estimating Equations
COPD	Chronic Obstructive Pulmonary Disease
PAM	Patient Activation Measure
ED	Emergency Department
PREMsT	Patient-Reported Experience Measurement
SHERPA	Sharing Evidence Routine for a Person-centered Plan for Action
NRRP	National Recovery and Resilience Plan
iNEST	Interconnected Nord-Est Innovation Ecosystem
CET	Comitato Etico Territoriale
ASOV	Area Sud-Ovest Veneto
Prot.	Protocol
